# Type 1 and Type 2 diabetes in the UK press: A diachronic corpus-based analysis

**DOI:** 10.1371/journal.pone.0348079

**Published:** 2026-04-30

**Authors:** Sara Vilar-Lluch, Dawn Knight

**Affiliations:** School of English, Communication and Philosophy, Cardiff University, Cardiff Wales, United Kingdom; Kwara State University, NIGERIA

## Abstract

News coverage of diabetes has been observed to promote diabetes stigma of both Type 1 (T1D) and Type 2 (T2D) diabetes, and to foreground individual responsibility over health outcomes. Adopting a corpus-based approach to discourse analysis, this study examines trends in media reporting around T1D and T2D in mainstream UK news media between 2020–2024. We consider: the linguistic representation of T1D/T2D; whether news reports include representations that could promote misunderstandings of T1D/T2D and contribute to perpetuating stigma; and whether language use aligns with recommendations in guidelines on diabetes communication. UK news coverage of T1D/T2D adopts medical and free will discourses while omitting references to social determinants of health (SDH) that can condition development and management of health conditions. While the medical discourse can help mitigate stigma, omitting references to SDH foregrounds individual agency and responsibility in adopting health-preventive behaviours while overlooking inequalities that may contribute to develop T2D or interfere with T1D/T2D management. Language recommended by the guidelines is increasingly adopted, but reduction in dispreferred language is less marked. References to diabetes ‘in general’, and references to diabetes in close context with unrelated conditions are common, leading to potential misunderstandings. Recommendations to improve communication include continuing the use of language recommended in diabetes guidelines and reducing uses of dispreferred language; referring to diabetes types to prevent misunderstandings; and taking special care when referring to other conditions to avoid inaccurate inferences of causality or similarity.

## 1 Introduction

### 1.1 Diabetes: a brief overview

In November 2024, the publication of new diabetes prevalence estimates by the NCD Risk Factor Collaboration (NCD-RisC, a global network of health scientists working on non-communicable diseases, ncdrisc.org) led to a global call to action and monitoring framework to address the soaring diabetes rates [1–2]: an estimated 828 million adults (aged 18 and older) lived with diabetes in 2022, an increase of 630 million from 1990 [3].

Diabetes is a chronic and serious condition that either involves an insufficient production of insulin (the hormone that regulates sugar in blood) by the pancreas, or an inability of the body to use the insulin effectively [4]. There are two main types of diabetes: Type 1 (T1D) and Type 2 (T2D). If untreated or ineffectively managed, diabetes can lead to premature death [4]. In 2021, 1.6 million deaths globally were directly related to diabetes [5], and in 2022, the number of adults with diabetes not receiving treatment was 3.5 times higher than in 1990 [3].

In 2023–2024, c.5.8 million people were estimated to live with diabetes in the UK, with a further 1.3 million people living with undiagnosed T2D, and an estimated 6.3 million people at risk of T2D [6]. These figures underscore the need for public understanding about the condition to obtain early diagnosis, mitigate risks and promote self-management and preventive behaviours.

### 1.2 Aims

This study considers news media coverage around diabetes in the UK over a period of five years (2020–2024). While news media plays an active role in shaping public understanding of health matters [7] and its potential to shape public opinions and political reality is well recognised [8–9], inappropriate communication around diabetes has been observed in news media platforms [10–12]. We adopt a corpus-based approach to examine:

(1) News media trends in T1D and T2D reporting in the UK press, focusing on patterns of linguistic representation;
(2) Whether language use in news media aligns with the one recommended in diabetes communication guidelines;
(3) Whether news media reporting about diabetes T1D and T2D reflects any misunderstanding of the conditions; and
(4) Whether the representations of T1D and T2D promoted in news reports could perpetuate stigma.

The next section provides the context to this study, underlining the importance of considering the language of diabetes care, particularly in news media. Section 3 describes the diabetes news corpus compiled for this study and the analytical approach adopted. Section 4 discusses analysis findings, followed by recommendations for media reporting in Section 5.

## 2 Background

### 2.1 Diabetes, stigma and communication

This study focuses on the two main types of diabetes: Type 1 (T1D) and Type 2 (T2D). T1D typically occurs in childhood or teenage years, and it involves insufficient insulin production, while T2D, the most common type, is commonly acquired in adulthood [4]. Causes of T1D are unknown and the condition is currently unpreventable [4]. T2D is associated with genetic and metabolic factors, and physical inactivity, overweight and obesity are identified as major risk factors [4]. Measures to prevent T2D, complications and premature death from both types are available [4].

Diabetes stigma is observed for both types [13–16]. T2D stigma is related to the social perception that T2D is a ‘lifestyle disease’ [13], a ‘moral failing’ resulting from lack of responsibility, self-negligence, overindulgence, laziness, or being overweight/obese [13,15,17], with T2D and obesity stigma being intertwined [18–19]. T1D stigma is identified by association with T2D, which may lead those individuals living with T1D wanting to distance themselves from those with T2D, reinforcing T2D stigma [14,16].

Parents of children with T1D and high insulin dependent individuals may also face stigma [16]. Other experiences of stigma include being blamed for inadequate diabetes management, social exclusion, or societal disbelief about T1D (among adults) due to identifications of T1D with childhood [14]. Stigma can lead to diagnosis concealment for both types, mismanagement, self-limiting behaviours, and distress [16]. Feelings of ‘shame, guilt, regret and hopelessness’, low self-esteem and low self-confidence are observed among individuals with T2D [13].

Media, including news and social media, popular culture and health campaigns, is identified as a main source of stigma (T1D, T2D) and associated with societal misconceptions [13–15]. People living with T1D and T2D diabetes have observed the media tends to report about ‘diabetes in general’, grouping the different types together [14,17]. Failure to distinguish between types promotes misunderstanding about causes and risk factors, and lack of awareness of T1D. T1D reportedly receives less media coverage than T2D and, when it receives coverage, this is often appraised as inaccurate by those living with T1D [14,17]. Media studies evidence the tendency to report on ‘diabetes in general’ and the focus on T2D when type is specified (e.g., [20–21]). Other media misconceptions include to exclusively associate T2D with poor diet and lack of physical activity, diabetes (in general and T2D in particular) with obesity, and a general disregard of the economic costs and emotional burden associated with both diabetes types [17].

Responding to the need for appropriate communication to promote T1 and T2 diabetes awareness and reduce stigma, there has been a proliferation of national and global initiatives focused on improving language use around diabetes. These initiatives include Diabetes Australia’s position statement [12,22], Diabetes Canada’s consensus statement [23]; the American Association of Diabetes Educators and American Diabetes Association’s consensus report [24]; the International Diabetes Federation’s ‘language philosophy’ [11] and recommendations from the Language Matters Foundation, whose guidance has been adopted by different countries, including the UK [25].

These guidelines favour removing language that connotes negative judgements, blame, criticism, disrespect (e.g., use of ‘diabetes’ in punchlines), high imposition directives or patronising attitudes. For instance, referring to people as ‘diabetics’ or ‘sufferers’ implicitly establishes diabetes as the main identity trait of an individual or place the person in a powerless position; the noun phrase ‘person living with diabetes’ is preferred instead [24]. Describing diabetes management as being ‘(non)adherent’ or ‘(non)compliant’ is discouraged; these adjectives are character attributes instead of behavioural descriptions and evoke moral judgements. Expressions like ‘managing’ diabetes or glucose levels are preferred to ‘controlling’ diabetes, since glucose levels may be influenced by factors beyond someone’s control (e.g., hormones) [12,23,24]. Dispreferred language use is reportedly still observed in the press (e.g., [26–28]) and is noted by individuals living with both types of diabetes as an ongoing problem, including in official health information [17].

### 2.2 News media and diabetes

News media has an impact on which medical conditions are of public interest, how medical conditions and those with the diagnoses are perceived and treated in society, and potentially influence policy and public expenditure. News media studies explain these effects through the processes of ‘agenda setting’ [8] and ‘framing’ [29]. ‘Agenda-setting’ refers to the transfer of prominent topics on the media to the public agenda, which conditions what is a matter of public interest, how topics are thought about, and public behaviour [8–9]. ‘Framing’ involves selecting aspects of the reality reported and making them more salient than others to promote a viewpoint, course of action or evaluation [29]. Reports about a medical condition can foreground its medical character (medical frame), the individuals’ agency to adopt health-promoting actions (behavioural or ‘episodic’ frame), or the societal structures that contribute at placing some individuals at a higher risk of developing a condition (societal or ‘thematic’ frame) [30–31]. Following Goffman [32], frames organise our experience by providing us with principles or rules of meaning attribution. This understanding of ‘frame’ broadly aligns with the concept of ‘discourse’ of the discourse analysis tradition, followed in this study. ‘Discourse’ is understood as language-in-use and as the frameworks that condition what and how we think and talk about phenomena [33]. Therefore, studying news media discourse requires examining both the linguistic choices used in news reports and the social practices enacted in these texts (e.g., the process of medicalisation, see [34]).

News coverage of diabetes (T1D and T2D) has consistently adopted a medical discourse and emphasised individual agency, while societal discourses are reported as deficient or largely neglected [20,21,28,35,36]. An increasing tendency to frame diabetes as a societal issue has been observed in the UK press [37]. More typically, news media defines diabetes as a medical problem and underscores individual responsibility over health outcomes by associating diabetes (particularly T2D) with specific lifestyles and emphasising individual behaviours as remedies (e.g., exercise, diet) (e.g., [20,35,36]). Maternal responsibility (and blame) is often emphasised in relation to developing diabetes (in general) in childhood [37]. Obesity is often represented as both risk factor and cause [35,37], particularly associated with T2D [20], while societal causes such as poverty, national economy, healthcare facilities or food industry regulations are often omitted (e.g., [20,37,38]).

S1 Appendix provides a non-exhaustive overview of studies of news media coverage of diabetes, indicating period examined, country, method used and focus of analysis. Many studies of news media coverage of diabetes have considered reports on diabetes in general, without explicitly distinguishing between types (e.g., [26,28,37,39]). When specific types are considered, a focus on T2D predominates (e.g., [35,36,38]). These studies tend to adopt content, thematic and frame analyses, which allow for identifying salient themes of, normally, small datasets (e.g., [20,21,35–38], see S1 Appendix). Studies focusing on media reporting around diabetes in the UK context are scarce (but see [37,38]). In these studies, diabetes is either addressed ‘in general’ [37] or as related to other conditions (e.g., [38], which considers news reports on cardiovascular diseases and T2D published in 2008 over an eight-month period). In this paper, we provide novel insights into the study of news media coverage of diabetes, specifically in the UK context, by: (1) Showing how corpus linguistics methods can be integrated into public health messaging and provide scalable results by presenting a comparative corpus-based diachronic analysis of T1D and T2D coverage in UK news media over 2020–2024 across ten mainstream UK newspapers, totalling over 9.9 million words; (2) Better revealing news media trends and reporting patterns than studies of smaller datasets; (3) Assessing whether news media (i) aligns to diabetes communication guidelines, (ii) reflects (mis)understandings associated with T1D/T2D, and (iii) contributes to stigma.

## 3 Materials and methods

### 3.1 The diabetes news corpus

We built, and analysed, a corpus of news articles about T1D and T2D published by UK news outlets in a five-year period, from 1^st^ January 2020–31^st^ December 2024 (the last complete year at the date of data collection) (see [54] for the Diabetes UK News Media corpus 2020–2024). News articles were published by the top ten UK national news outlets (paper and online publications) according to reported circulation [40–42]: *The Guardian*, *The Times*, *The Sun*, *Metro*, *Daily Mail*/*Mail Online*, *The Telegraph*/*Daily Telegraph*, *Daily Mirror*, *i*/*i Newspaper*/*The i Paper*, *Daily Express* and *Daily Star*. Articles were retrieved from the Nexis Lexis database, which provides access to a comprehensive range of UK newspapers, using the search queries *“diabetes type 1” OR “type 1 diabetes”* and *“diabetes type 2” OR “type 2 diabetes”.*

Applying Nexis Lexis’s inbuilt filters, articles were filtered by region and language (‘United Kingdom’, ‘English’) and news platform (see above), grouped by ‘moderate similarity’ (an inbuilt feature that automatically analyses documents and groups duplicates and texts that share a great amount of content (e.g., online updates, newswires), retrieving the most recent version only); newswires, financial news and obituaries were excluded. Boilerplate text (date and time of publication, reporter’s name, news platform, text added by Nexis Lexis, e.g., “Body”, “end of article”) was removed using a Python script (developed by Michail Papanikolaou). All Nexis Lexis news articles documents share the same template. The script keeps all the text in-between “Body” and “end of the article”, removing all the text preceding (and including) “Body”, which marks the start of the news article, and all the text following (and including) “end of the article”. Articles were compiled in.txt files, classified by year, news outlet and diabetes type. We built two independent sub-corpora: diabetes type 1 (T1D) and type 2 (T2D), as detailed in Table 1. Combined, the corpora comprise a total of 9,673 news articles and total around 9,977,249 words (11,446,354 tokens).

**Table 1:** Overview of the diabetes news corpus.

	T1D sub-corpus			T2D sub-corpus		
Year	Articles	Words	Tokens	Articles	Words	Tokens
2020	182	147,554	182,579	1,410	1,510,918	1,741,757
2021	223	376,108	427,694	1,193	1,363,068	1,559,682
2022	235	307,606	352,106	1,161	1,140,245	1,296,670
2023	270	205,018	237,599	1,784	1,634,145	1,882,984
2024	278	290,944	335,996	2,937	3,001,643	3,429,287
***Total***	*1,188*	*1,327,230*	*1,535,974*	*8,485*	*8,650,019*	*9,910,380*

### 3.2 Analytical approach

This paper adopts a corpus-based approach to discourse analysis (see [43]). Corpus approaches are software assisted, allowing for the detailed analysis of large collections of texts (corpora). For this study, we consider the collocates for the term ‘diabetes’ in each sub-corpus (Table 1). Collocations are words that tend to co-occur more frequently than what would normally be expected, based on frequency of co-occurrence and association measures [44]. Analyses of collocation suggest that a word’s meaning is defined, to some extent, by the words typically used with it (its *collocates*). Examining the collocates of ‘diabetes’ in each sub-corpus can illuminate popularised understandings and evaluations of T1D and T2D.

Collocates were retrieved using the Word Sketch functionality of the online corpus analysis tool Sketch Engine. Collocation strength is calculated with logDice, with 14 as maximum theoretical value (absolute co-occurrence) and negative values indicating that the co-occurrence is not significant [45]. The higher the logDice values, the higher the typicality of the co-occurrence. Word Sketch calculates collocates according to their grammatical function, thus providing insight into their context of use and allowing for an easier identification of patterns. Further examination of context of use was supported by the Concordance tool, which displays all instances of the word of interest in its lexical context and allows for qualitative examination of its use, facilitating pattern identification and interpretation of meaning.

We have considered collocates for the following grammatical structures in the two sub-corpora: pre-modifiers of ‘diabetes’, verbs with ‘diabetes’ as direct object, verbs with ‘diabetes’ as grammatical subject, ‘diabetes’ followed by the coordinating conjunctions ‘and’/‘or’, the prepositional phrase ‘with diabetes’, and the relational clause ‘diabetes is’. These grammatical structures allow for a focus on actions associated with diabetes, explicit descriptions of the condition (both as verbal clause and as nominal clause, adjective + noun), social actors characterised with diabetes (post-modifying ‘with’ prepositional phrase), and entities associated with diabetes (with ‘and’/‘or’ conjunctions).

The top 10 collocates of ‘diabetes’ ranked by collocation strength for each grammatical structure were considered for each year and sub-corpus (summary tables provided in S2 Appendix and S3 Appendix). Collocates were manually classified into the following themes to assist pattern identification: dispreferred language and language recommended (which corresponds to the expressions discouraged and recommended in diabetes guidelines respectively, see Section 2.1 and [11,12,22–24]); diabetes types (i.e., T1D, T2D, gestational or general references); references to other conditions (i.e., any other medical condition, including both physical and mental health conditions); verbs: diabetes onset; and verbs: having diabetes, which focus on the start of the condition and on living with diabetes respectively (see Section 4.2 Table 3 and Section 4.3 Table 4). Thematic classification of the collocates and interpretation of themes was assisted by concordance checks.

## 4 Diabetes news coverage in the UK press

### 4.1 Overview

T2D consistently receives more news media attention than T1D in the UK press (Table 2), aligning with press coverage in other contexts (e.g., [20,35]). While both T1D and T2D news coverage increase over the 5-year period, the sharp increase observed for T2D in 2023–2024 is not mirrored by the T1D coverage. The growth in diagnoses during 2023–2024 [6] could partly account for the rise in coverage. Given that diabetes diagnoses have risen for both types [6,46], the increasing T2D coverage might be influenced by other factors (e.g., popular association of T2D with obesity).

**Table 2:** Diabetes types and use of ‘language to avoid’.

Word	T1D corpus (1,327,230 words)		T2D corpus (8,650,019 words)		2020		2021		2022		2023		2024	
	Freq.	RF	Freq.	RF	RF - T1D	RF - T2D	RF - T1D	RF - T2D	RF - T1D	RF - T2D	RF - T1D	RF - T2D	RF - T1D	RF - T2D
*‘Diabetic’* (noun)	259	19.51	179	2.07	24.40	2.71	13.56	2.05	17.55	2.54	30.73	2.20	18.90	1.50
*‘Diabetic’* (adj.)	77	5.80	275	3.18	5.42	4.63	3.72	1.91	4.88	3.68	7.32	4.71	8.59	2.00
*‘Sufferer’*	176	13.26	530	6.13	11.52	8.74	7.18	6.53	10.40	6.31	28.29	6.98	14.44	4.10
*‘Patient’* (noun)	1639	123.49	1041	12.03	93.53	12.58	141.45	16.36	87.45	12.45	136.09	12.12	144.70	9.59

*Relative frequencies (RF) are calculated with a base of 100,000. Frequencies for the whole T1D and T2D sub-corpora include the total frequency of the term (aggregated value across 2020–2024).

** The counts of ‘diabetic’ (adj.) include: ‘be + diabetic’, ‘become + diabetic’, and ‘diabetic’ as pre-modifier of nouns referring to human actors (e.g., *adult, child, daughter*).

Table 2 features uses of dispreferred language to refer to people living with diabetes across 2020–2024, specifically, ‘sufferer’, ‘patient’ (noun), and ‘diabetic’ (noun and adjective, see [12,24]). ‘Patient’ and ‘sufferer’ are used at higher rates in the T1D sub-corpus than in the T2D sub-corpus, suggesting that news coverage of T1D tends to adopt a stronger medical discourse; dispreferred language use does not show any decline. Uses of dispreferred/recommended language and discourses adopted are explored in sections 4.2 and 4.3.

### 4.2 T1D

#### 4.2.1 Reporting tendencies.

News coverage in the corpus increasingly adopts language recommended in diabetes communication guidelines (theme 2 Table 3). Uses of the collocates *manage*, *condition* and *live* / *living* [with diabetes] increase over the five-year period. Dispreferred language, including explicit references to *disease* to refer to diabetes and related conditions, references to *uncontrolled* diabetes and *control* to refer to management strategies, and references to individuals as *patients*, has fallen at a comparatively slower rate.

**Table 3:** Collocates for “diabetes” in T1D sub-corpus classified by theme.

Themes	Collocates	2020	2021	2022	2023	2024	Themes	Collocates	2020	2021	2022	2023	2024
		RF*	RF	RF	RF	RF			RF	RF	RF	RF	RF
1. *Dispreferred language*	control	4.07			7.32	2.06	5. *Verbs - diabetes start*	diagnose	3.39	1.33	1.3	1.95	
	patient	0.68	1.33	0.65		1.03		develop	3.39	3.19	1.3	1.46	3.78
	disease	4.07	1.33			2.06		cause	6.1	2.13	1.3	1.95	2.75
	uncontrolled			0.98				occur	2.03	0.53	0.98		2.41
	treat			1.3				start	1.36	1.33			
	*Total*	8.81	2.66	2.93	7.32	5.16		trigger		1.06			
2. *Language recommended*	manage	2.71		2.28	9.76	5.5		appear		0.53			1.37
	condition	6.1	2.13		4.88	4.81		delay			0.98		
	live/living	2.03	0	0.65	0	7.22		get			1.63		
	*Total*	10.8	5.85	2.93	22	17.5		emerge			1.3		
3. *Diabetes types*	Type 1	0	0.8	0	3.9	0		begin					1.03
	Type 2	2.03						*Total*	16.27	10	8.79	5.36	11.34
	neonatal	2.03					6. *Verbs - having diabetes*	tackle	1.36				
	juvenile	1.36	0.53					present	1.36				
	monogenic		5.85					control	1.36			7.32	2.06
	maternal		1.6					have	18.3	9.57	13.33	29.27	9.97
	gestational		1.6	2.93				manage	2.71		2.28	9.76	5.5
	autoimmune			0.98	1.46			affect	1.36	0.8	1.63		
	*Total*	22.4	10.37	7.15	5.37	4.47		live	2.03				
4. *Other conditions*	asthma	2.03	0.8					pre-exist		0.53			
	glaucoma	1.36						accelerate		0.53			
	condition	2.71		1.63	3.41	1.37		grow		0.8			
	arthritis	1.36						lead		0.53	0.98		
	disease	3.39	2.92	2.28	10.2	9.97		cause		0.8	4.23	4.88	2.06
	level	1.36						destroy		1.06			
	cancer	1.36				1.72		reverse		0.53			
	type	1.36				1.37		live/living		3.7	0.65	7.32	7.22
	sclerosis		1.06					treat			1.3		
	insufficiency		0.8					change			1.3		
	epilepsy		0.53					rise				1.46	
	pressure		0.8					need				3.41	
	obesity		0.8	1.95		1.37		use				1.95	
	lupus		0.53					monitor					1.03
	type		1.06					remain					1.37
	pancreas			0.98									
	arthritis				1.95	1.72							
	depression				1.46								
	thyroid					1.03							
	*Total*	14.9	8.24	6.83	17.1	18.6		*Total*	28.48	19	25.7	65.37	29.21

*Relative frequencies (RF) are calculated with a base of 100,000.

Explicit references to diabetes types decrease over the years (theme 3 Table 3). Of the 5,258 occurrences of *diabetes* in the T1D sub-corpus, 2,291 occurrences (43.57%) do not specify type. A total of 2,428 (46.18%) uses of *diabetes* include a reference to Type-1 (*Type one*, *Type-1*) as pre-modifier, and the remaining 539 occurrences (10.25%) include references to other types (*Type-2*, *gestational*, *monogenic* (diabetes caused by mutations of a single gene) or references to Type-1 as *juvenile diabetes*, 28 instances). These observations support concerns raised by individuals with T1D about the tendency of the press to refer to diabetes ‘in general’ [14].

References to specific medical conditions collocating with ‘diabetes’ (theme 4, Table 3) decreased during 2021–2022 but increased from 2023–2024. As with T1D, some of these conditions are autoimmune and co-occurring references may be based on this similarity (example 1). However, references to medical conditions are not always based on aetiology (causes) similarities and do not always specify diabetes type, which may contribute to T1D misunderstanding (see section 4.2.2).

(1) These people may also be at higher risk of autoimmune diseases […] such as rheumatoid arthritis, lupus, type 1 diabetes, and multiple sclerosis. (*Daily Mail*, 2023)

Themes 5-6 include verbs to report diabetes onset (theme 5, e.g., *occur*, *start*, *appear*) and living with diabetes, including actions to manage the condition (theme 6, e.g., *control*, *manage*, *monitor*) and actions attributed to T1D, mostly denoting how it affects people’s lives (theme 6, e.g., *affect*, *cause*) or how it evolves (theme 6, e.g., *grow*, *accelerate*) (Table 3). Verbs describing living with diabetes are more frequent than those describing the onset across the five years, suggesting a higher focus on reporting management strategies and the impact of T1D on people’s health than on causes or triggers, potentially reflecting that the exact cause of T1D is unknown.

All the verbs identified for theme 6 (Table 3) attributed to a human subject denote agency or control (*manage*, *control*, *monitor*) except for *have*, which construes living with diabetes as a possessive attributive relation (‘…have diabetes’). *Need*, a relational attributive verb not connoting agency or control on its own, is used to describe self-management strategies by reporting the need of “daily injections of insulin” to control blood glucose levels in all cases (year 2023, Table 3). The salient use of verbs conveying control depicts T1D as a condition that requires high individual responsibility to avoid or reduce its negative effects. Reports on effects tend to specify diabetes type (especially with *cause*, e.g., “Type 1 diabetes causes the level of glucose (sugar) in your blood to become too high.”, *The Sun,* 2022) and may include figures to highlight its prevalence (especially with *affect*, e.g., “Type 1 diabetes affects 400,000 people in the UK”, *Daily Mail,* 2022). In other instances, however, the negative impact is attributed to diabetes ‘in general’. For example, from the 8 verbal clauses ‘diabetes leads to’, only one specifies type (T1D), the rest constitute generalisations (example 2).

(2) Every week, diabetes leads to more than 770 strokes, 590 heart attacks and 2,300 cases of heart failure. (*Daily Mail*, 2023)

#### 4.2.2 Medicalisation of news media.

News media coverage of T1D adopts, primarily, a medical discourse, evidenced by the use of medical terminology to describe the condition, complications associated with it and possible causes (Table 3, themes 1-4, and S2 Appendix). T1D is construed as an individual medical problem, and those individuals with the condition are presented with the responsibility to take control of their health (see verbs collocating with ‘diabetes’ as direct object, S2 Appendix or theme 6 Table 3). Table 3 features references to specific diabetes types, sometimes used to problematise popular yet misleading references to T1D (e.g., *juvenile diabetes*, theme 3, which promoted the erroneous association of T1D with young people exclusively, see example 3), and descriptions that reflect current medical understanding (e.g., characterisations of T1D as an “autoimmune” condition, theme 3).

(3) Type-1 is sometimes referred to as juvenile diabetes, but the term (sic.) regarded as outdated because the condition can develop at any age. (*Daily Mail*, 2020)

Reports of causes are relevant for the dissemination of knowledge around T1D. Causality may be expressed explicitly (e.g., using the verb *cause*, see Table 3, theme 5) or it may be inferred from the syntactic structure of the sentence. Inferences of causality (and similarity) are possible when T1D (or diabetes ‘in general’) are preceded or followed by other medical conditions (i.e., apposition relation), or the “and” conjunction is used (i.e., coordination relation) (see Table 3, theme 4 and example 4). Example 4 illustrates how causality and similarity can be inferred through coordination (“…and diabetes”): impaired blood circulation can be inferred as leading cause of diabetes, and diabetes is likened to a heart/circulatory condition through the identifying relational process ‘including’.

(4) This helps to move blood […] and when it stops working it can lead to a range of heart and circulatory conditions, including high blood pressure and diabetes. (*The Telegraph*, 2021)

These inferences can generate or promote misunderstandings (in example 4, that diabetes is a heart/circulatory condition, although this is not the case). Frequent references to different conditions in close context may allow to transfer negative evaluations (e.g., regarding risk, individual responsibility, health outcomes) commonly ascribed to one condition to the other one by inferred association. For instance, example 5 reports on viral infections as causes of T1D and compares it to cervical cancer and multiple sclerosis. The potential viral cause of T1D and multiple sclerosis is reported in scientific literature (e.g., [47–48]). The recurrent co-occurrence of ‘diabetes’ with *cancer*, *multiple sclerosis* and other conditions (Table 3) in different contexts can contribute to generate erroneous associations by repeatedly reading these conditions together.

(5) […] researchers have demonstrated that both Type 1 diabetes and cervical cancer are triggered by a viral infection […] And so too multiple sclerosis […] (*The Telegraph*, 2024)

Explicit causal attributions (i.e., the use of the verb *cause* with diabetes as direct object, 33 occurrences) identify T1D with viral and biological factors (e.g., *gene mutation*, *bacterial infections*) or explicitly challenge popularised beliefs (e.g., *Covid-19* as cause). Societal factors as potential causes are largely absent, although explicit references to “socioeconomic” factors feature 7 times. Other causal factors explicitly mentioned in the sub-corpus (collocates for factor*, 151 occurrences) include references to environment (*environmental*, 10 occurrences), individual biology (*genetic*, 7 occurrences) and lifestyle (*lifestyle* 13 occurrences, *obesity* and *diet*, 4 occurrences each), the latter of which is dissociated from T1D (example 6). Causal explanations may establish a direct opposition between T1D and T2D, which is explicitly associated with lifestyle choices (in example 6, lifestyle, diet and obesity are mentioned in relation to T2D). While these descriptions contribute to dispel misunderstandings of T1D, they may foster T2D stigma.

(6) Unlike the more common type 2 diabetes, which is linked to lifestyle, diet and obesity, type 1 diabetes is caused by genetics and environmental factors. (*The Times*, 2021)

### 4.3 T2D

#### 4.3.1 Reporting tendencies.

Uses of ‘language recommended’ in T2D reporting slightly increased over the period examined, but as for T1D, dispreferred language use remained stable (Table 4, themes 1-2). References to T2D management (*manage*, preferred to *control*) are increasingly used: while instances of *manage* were not identified among the collocates for the 2020 sub-corpus, all the other years include references to *manage* (Table 4, theme 2). Explicit references to control (*control*, *uncontrolled*), which is discouraged in the guidelines, remained relatively stable across the years (Table 4, theme 1). Descriptions of T2D as a *condition* individuals *live with* (recommended in the guidelines) are increasingly used alongside more medicalised identifications of T2D as a *disease* (identified as dispreferred language in the guidelines) (Table 4, themes 1-2, and S3 Appendix, collocates for ‘diabetes is’). Sometimes T2D is identified as a *crisis* (Table 4 theme 1, 2020). These portrayals explicitly refer to T2D and to T2D in conjunction with obesity (e.g., “the obesity and diabetes crisis”; “Type 2 diabetes is an urgent public health crisis”, *Daily Mail* 2020). References to people with T2D as ‘patients’ (noted as dispreferred language in the guidelines) remain consistent, but the corpus also features generic references throughout, often depicting adult population (e.g., *people*, *adult*, *woman*, *man*, all of which would adhere to guidelines recommendations) (S3 Appendix, collocates for ‘with diabetes’).

**Table 4:** Collocates for “diabetes” in T2D sub-corpus classified by theme.

Themes	Collocates	2020	2021	2022	2023	2024	Themes	Collocates	2020	2021	2022	2023	2024
		RF	RF	RF	RF	RF			RF	RF	RF	RF	RF
1. *Dispre* *ferred language*	disease	3.84	1.03	2.54	1.35	0.43	5. *Verbs – diabetes start*	develop	6.55	4.04	5.7	4.83	3.63
	uncontrolled	0.53		0.79	0.31	0.33		diagnose	3.24	5.94	3.77	1.22	1.8
	patient	0.86	0.51	0.61	1.71	0.8		get	1.92				
	crisis	0.46						occur	1.19	0.95	0.88	0.61	0.67
	treat		1.32	1.58	3	3.16		link	0.79				
	control		0.59		1.04	0.63		cause		1.47		1.22	
	*Total*	5.69	3.45	5.52	7.41	5.35		trigger		0.51			
2. *Language recom* *mended*	live/living	1.72	2.64	2.54	3.85	2.97		miss		0.51			
	condition	1.59	1.61	4.21	1.35	1.4		prevent			0.96		
	manage		1.25	1.23	1.77	1.43		*Total*	13.69	13.4	11.3	7.88	6.1
	*Total*	3.31	5.5	7.98	6.97	5.8	6. *Verbs – having diabetes*	reverse	2.65	3.01	3.07	1.41	1.8
3. *Diabetes types*	Type-2	5.56	4.25	2.28	2.57	3.93		have	13.83	15.9	13.5	7.96	5.26
	gestational	1.06	6.53	2.19	3	1.57		stop	0.93				
	Type-1	0.66						keep	1.06				
	adult	0.73						cause	0.93	0.73	1.84	1.29	0.47
	undiagnosed	0.79		0.61		0.23		put	2.38			0.98	0.57
	full-blown	0.6	0.51					double	0.728	0.88			0.27
	monogenic		1.32					face	0.794				
	maternal		0.29					increase	0.86	1.17	0.44	0.31	
	diabetes			0.53				go	1.986				0.43
	prediabetes			1.23				live / living	1.721	2.64	2.54	3.85	2.97
	*Total*	9.4	12.9	6.84	5.57	5.73		treat		1.32	1.58	3	3.16
4. *Other conditions*	obesity	7.48	6.38	8.51	6.61	0.67		manage		1.25	1.23	1.77	1.43
	disease	17.61	11.88	21.57	18.42	2.07		affect		0.73	0.88	0.61	0.6
	pressure	7.74	5.43	7.89	5.69	4.36		rise		0.51	0.53	0.37	0.37
	cancer	5.56	6.38	6.75	8.75	9.49		control		0.59		1.04	0.63
	stroke	2.52	1.25		2.51	1.9		struggle			0.44	0.43	
	hypertension	1.79	1.1	1.84		1.5		battle					0.17
	problem	1.92						remain					0.3
	dementia	1.32	1.17	1.49									
	condition	1.06	1.54	2.28	1.59	1.53							
	cholesterol		1.1			1.2							
	attack		1.03			1.2							
	depression			0.18	1.41	1.67							
	*Total*	47	37.26	50.51	44.98	25.59		*Total*	27.87	28.7	26.1	23	18.4

References to diabetes type are recurrent. Common characterisations of T2D include being *uncontrolled*, *full-blown*, *undiagnosed* (Table 4, theme 3). These references portray T2D as a looming risk that may go unnoticed until it is too late, and indeed it may be missed by clinicians (*miss*, Table 4 theme 5, all occurrences with health professionals as grammatical subject).

News coverage of T2D often refers to other medical conditions in close context with T2D (Table 4, theme 4; S3 Appendix, ‘and/or’ grammatical pattern). Conditions mentioned alongside T2D are depicted as risk factors or triggers of T2D (*obesity*) or associated with T2D (e.g., *hypertension*, *stroke*, [high blood] *pressure*, [heart] *attack*, *cholesterol* – see example 7, where a causal relation can be inferred through ‘linked’).

(7) Rising levels of type 2 diabetes are linked to an increase in rates of depression. (*The Times,* 2022).

*Depression*, *dementia* and *cancer* are increasingly discussed in conjunction with T2D. The connection between cancer and T2D mostly relies on *obesity* as common risk factor. For instance, in 2020 (when T2D was explicitly identified as *crisis*), the 84 occurrences of the collocation ‘diabetes and/or cancer’ (S3 Appendix) include 22 explicit mentions of obesity as risk factor, 19 references to diet (to reduce fat/sugary food) and 12 references to (in)activity (to avoid a sedentary lifestyle). The link between obesity and T2D is reinforced across the years (Table 4, theme 4, see also Table 6). The 2024 sub-corpus does not include *obesity* among the top ten collocates for the ‘…and diabetes’ grammatical pattern; instead, it features *Wegovy*, a medicine employed for weight management similar to Ozempic, used to manage T2D (S3 Appendix). Out of the 37 occurrences of *Wegovy*, 35 explicitly refer to overweight (*weight loss*, *obesity*, *weight management*, *obese*).

Descriptions of living with T2D predominate over accounts of T2D onset across the years (Table 4, themes 5-6). Although Table 4 includes references to remission (*reverse*, *put* [into remission], *stop*), the emphasis is on prevalence (*affect*, *increase*, *rise*, *double*), management (*control*, *manage*, *treat*), and the effects of T2D on individuals’ health (*cause*, [difficulties individuals] *face*). While the most recurrent portrayals of living with T2D include non-evaluative verbs (*have*, *live*), occasionally the experience of T2D is depicted as highly difficult (*struggle*, *battle* in 2023 and 2024).

The trends observed in T2D news coverage portray T2D as a condition involving high (health) risks, increasingly widespread and occasionally unnoticed, but associated with factors under individuals’ control (being active, healthy diets). Emphasising the risks associated with T2D can foster social alarm, while the capacity (and responsibility) to mitigate the risk is placed on the individuals’ free will.

#### 4.3.2 Lifestyle and individual responsibility.

News media reporting of causes, management and effects of T2D adopts two contrasting discourses: a discourse of ‘free will’ or appellation to individual responsibility, especially adopted to describe causes; and a medical discourse, especially adopted to describe management strategies (see Table 5, featuring the collocation patterns “cause diabetes”, “manage/control diabetes”, and “diabetes causes”). News media primarily associates T2D with lifestyle choices, including physical inactivity, diet and obesity/overweight across all years examined (e.g., the reference to ‘bad diet’ in example 8; Table 5). Other reported (potential) causes include Covid-19 (during 2021–2022), stress (*stress*, *cortisol*) and *blue light* exposure (Table 5). On one occasion (example 9), T2D is associated with a combination of factors, including genetics.

**Table 5:** Causes, management strategies and effects of T2D.

Theme	Collocation	2020	2021	2022	2023	2024
Causes	*‘cause diabetes’*	obesity/excess fat (4); diet [alcohol] (1); sugar (1) [denied as cause]; bacteria (1) [denied as cause]; ‘chemical reactions’ (1); cystic fibrosis (2)	Covid-19 (7); obesity/excess fat (4 + 6 contesting it)	obesity/excess weight (2); stress (3); sugar (3); Covid-19 (2)	obesity/excess fat (4); sugar (3 + 3 myth); lifestyle [including diet] (2)	obesity/excess fat (1); sugar (1); inflammation (1); lifestyle [including diet/alcohol] (6); blue light (2); cluster of factors [including environmental, genetic] (1)
Management	*‘manage diabetes’*	diet (5); lifestyle (1)	diet (6); medication (2)	medication (5); diet (3)	medication (18); lifestyle (1); diet (3)	medication (24); lifestyle [including diet] (13); social determinants (1)
	*‘control diabetes’*	diet (8); lifestyle (1)	diet (4); lifestyle (2)	medication (1)	medication (8); lifestyle (1); diet (1)	medication (10); diet (2)
Effects	*‘diabetes causes’*	high levels of sugar in blood (6); death (20); ill health (2); poor living conditions (2); erectile dysfunction (1)	nerve damage (1); [general health] ‘complications’ (1); inflammation (1); ‘weight fluctuations’ (1); ‘unstable blood sugar levels’ (1)	high blood sugar (4); cardiovascular problems (3); sight loss (1); hair loss (1); nerve damage (1); erectile dysfunction (2); gangrenous limbs (1)	skin problems (1); high levels of sugar in blood (6); unhealing wounds (1); death (1); life shortening complications (3); depression (3) [link unclear]	long-term illness (1); nerve damage (2); high levels of sugar in blood/urine (4)

(8) AROUND seven in 10 of all cases of type 2 diabetes are caused by bad diet. (*Star*, 2023)
(9) Type-2 diabetes is caused by a range of factors from genetics to where the body stores fat, with our food environment also playing a significant role. (*Daily Mail*, 2024)

Causal attributions of T2D to lifestyle may evoke negative moral evaluations of those individuals who develop T2D. In example 8, the evaluative inference is triggered by “bad [diet]”. The assumption is that lifestyle is an individual choice; the lack of references to socioeconomic or health-related factors that may condition lifestyle decisions reinforce the assumption of free will and the (negative) moral evaluation evoked.

Management strategies increasingly refer to medication and less to lifestyle (Table 5). In the 2020 sub-corpus references to medication do not feature for the collocates considered. In the 2021 sub-corpus, 35.29% of the collocates “manage diabetes” (six out of 17 occurrences) refer to diet whereas only 11.76% (two occurrences) refer to medication. In contrast, in 2023 only 10.34% and 3.44% of occurrences (three and one occurrences out of 29) refer to diet and active lifestyle, whereas 62% (18 occurrences) refer to medication. In 2024, more than half of references to management strategies refer to medication (24 out of 43 occurrences of *manage*, and 10 out of 19 occurrences of *control*). The predominance of references to medication portrays T2D as a medical condition that cannot be prevented or managed with lifestyle choices only, which can contribute to minimise negative moral judgements.

References to obesity in the T2D corpus are prevalent and increase over the years, with references to *obesity epidemic* / *crisis* occurring in conjunction with ‘diabetes’ (Table 6). Although the causal association between obesity and T2D is sometimes contested (example 10), obesity is often depicted as a cause of T2D (example 11), or even as the direct cause of the increase in T2D prevalence (example 12). The absence of references to socioeconomic and health-related factors leading to obesity/overweigh contributes to convey obesity as a matter of choice and, by inference, T2D.

(10) …diabetes is not caused by obesity but by being too heavy for your own body (*The Guardian,* 2021)
(11) Type 2 diabetes is usually caused by obesity. (*Daily Mail,* 2023)
(12) Britain’s obesity epidemic has led to soaring levels of type 2 diabetes (*Daily Mail*, 2020)

T2D is described as the cause of ill health (Table 5, direct object of ‘diabetes causes’), including high levels of sugar in blood and cardiovascular diseases, nerve damage, erectile dysfunction, hair loss, sight loss, and general ill health.

**Table 6:** References to obesity and diabetes.

	2020	2021	2022	2023	2024
‘Obesity’	1,675	1,221	1,570	2,385	3,768
	RF = 110.86	RF = 89.58	RF = 137.69	RF = 145.95	RF = 125.53
‘obesity epidemic’	47	37	64	71	89
‘obesity epidemic’ + ‘diabetes’/’type 2 diabetes’	19	7	4	0	0
‘obesity crisis’	64	34	58	67	94
‘obesity crisis’ + ‘diabetes’/ ‘type 2 diabetes’	8	2	2	3	0

## 5 Concluding discussion

### 5.1 Summary of main findings

UK news media shows competing medical and free will discourses in T1D and T2D reports, and a gradual increase in the use of language recommended by diabetes guidelines, albeit the latter is not mirrored by a similar decrease in dispreferred language. Although the newspaper articles include references to diabetes types, references to diabetes ‘in general’ are common, supporting reported concerns of individuals living with T1D/T2D [13,17] and echoing findings of news media studies in other contexts (e.g., [20] in New Zealand). In the Diabetes UK News Media Corpus, general uses of ‘diabetes’ occur when reporting risks common for both diabetes types, but also stand metonymically for one type, ultimately relying on readers’ ability to discern the type of diabetes discussed (see example 10).

As observed in previous studies of diabetes representation in news media (e.g., [20,21,28,35,36]), medical discourse is also prominent in the corpus examined. In the Diabetes UK News Media Corpus, the medical discourse is used in articles that mention both diabetes types but is particularly common for T1D. The medical discourse alludes to medical causes and medicines as management strategies. It is also enacted by referring to T1D and T2D in the context of other medical conditions, either by portraying some observed similarity (specially for T1D), or by presenting diabetes as the causal factor (specially for T2D). Causal relations or common risk factors are sometimes explicitly stated. Other times, however, associations between medical conditions appear unclear, which can lead to erroneous inferences of causality or evaluations by association.

The medical discourse can contribute to reduce moral blame and stigma associated with T2D, popularly regarded as a consequence of unhealthy diet and sedentary lifestyle. While the medical discourse is adopted, discussions around the social determinants of health (SDH) [49] that may impact on diabetes development and management were not frequently featured in the articles. SDH are conditions in which people are born or live that, although not directly related to health, affect individuals’ health, such as income, food security, education or social inclusion [49–50]. Omission of societal factors is common in news media reporting of diabetes (e.g., [20,37,38]). Overlooking SDH ultimately contributes to depict diabetes (both types) as an individual problem, and diabetes management as an individual responsibility. The collocation analysis did not reveal any discussion around the affordability of healthy diets and the ongoing UK food crisis [51–53], neither did it include references to the affordability of adopting active lifestyles across demographics, or debates on economic policies to promote healthy eating. Instances referring to the economic and emotional burden of living with diabetes (T1D or T2D) have not been observed in the analysis.

The medical discourse competes with an underlying discourse of free will, particularly observed for T2D. References to lifestyle and diet do not predominate among the management strategies identified in the corpus, contrasting with previous studies which reported recurrent associations of T2D with individual behaviours and lifestyle changes as remedies (e.g., [20,35,36]). However, lifestyle (including dietary habits) features in descriptions of causes leading to T2D and in associations of T2D with obesity/overweight. The absence of discussions around factors leading to obesity/overweight (e.g., genetic, ill health, socioeconomic) can contribute to reinforce its association with over-indulgence; by inference, T2D is implicitly depicted as an individual moral failure. T1D is not associated with negative moral judgements, and autoimmune and genetic causes are emphasised, sometimes including explicit contrasting descriptions with T2D. Verbs conveying control are common in T1D reporting. These verbs depict T1D as requiring high individual responsibility, evoking positive assessments of those that manage it successfully. However, failure to refer to factors that hinder T1D management implicitly blames the individuals (or their parents/carers) for health complications.

Overall, this analysis has shown that UK news media reporting on T1D and T2D reflects competing societal discourses on health and wellbeing. The medical discourse promotes current scientific understanding and avoids moral blaming. The avoidance of discussions around societal structures (societal frames) in reporting management strategies and (T2D) causes, however, places health-preventive actions in the individual responsibility exclusively, overseeing prevalent inequalities that may contribute to develop T2D, or that interfere with T1D and T2D management.

### 5.2 Health communication recommendations

Recommendations for news media reporting around diabetes (type 1 and type 2) emerging from this study include:

- Continuing the use of the language recommended in diabetes guidelines (see [11,12,23,24]) and reducing the uses of dispreferred expressions. Diabetes guidelines recommendations reflect the linguistic choices commonly preferred by individuals with lived experience.
- Making explicit reference to diabetes types should be the norm to avoid misunderstandings. If readers lack diabetes health literacy, general descriptions risk relying on stereotypes or inaccurate pre-existing beliefs. For general references to diabetes, or references that are meant to include both T1D and T2D, it is suggested to explicitly state this use –e.g., ‘diabetes (in general)’, ‘diabetes (types 1 and 2)’.
- Adopting a wholistic perspective towards diabetes which acknowledges both medical and societal factors can help prevent individual blaming. Reflecting on the social determinants of health [49] in news media may help palliate diabetes stigma when triggered by societal perceptions of moral failure.
- Taking special care when referring to diabetes (in general or specifying type) in close context with other medical conditions, especially when the purpose of this juxtaposition is not specified or cannot be retrieved from the surrounding context, to avoid unintended inferences of causality or similarity between the conditions. Since many medical conditions still present social stigma, unclear references to different conditions in close context may contribute to trigger (negative) evaluations of one condition by association with the others.

Insofar as news media is a main provider of health-related information for the public [7], news platforms active engagement with the considerations above can contribute to rise better awareness and understanding of T1D/T2D among their readerships.

### 5.3 Limitations and further research

Corpus-based discourse studies of news media can provide scaled up and diachronic insights into reporting trends, making it possible to identify patterns and changes in reporting which can reflect societal changes in the understanding of the health conditions discussed. Results of this analysis, however, cannot expand beyond the Diabetes UK News Media Corpus under consideration or explain audience reception of the texts. This includes considerations about the data selection (news media platforms included, timeline considered) and search query used to build the corpus (for this study, with references to type 1 and type 2 diabetes exclusively, excluding references to gestational diabetes, according to research objectives). For example, the Diabetes UK News Media Corpus includes the top ten UK national newspapers, meaning that it does not account for potential differences in reporting patterns across national and regional newspapers. Differences across national and regional newspapers reporting on health matters have been observed for mental health in the UK context [55], suggesting that this could also be the case for physical conditions. While the Diabetes UK News Media Corpus includes both tabloid and broadsheet newspapers (traditionally regarded as ‘popular’ and ‘serious’ press respectively), the analysis has not considered potential differences in reporting trends across the two publication types. Differences in reporting around health matters across tabloids and broadsheets, however, have been observed in corpus-based studies of British news media. For example, in comparing representations of autism in British tabloids and broadsheets, [56] shows how tabloids feature more stereotypically negative portrayals of the condition. The present study could be refined by examining whether all news outlets adopt equally the language recommended in diabetes guidelines or whether some platforms show higher trends of dispreferred language, and whether the medical and free will discourses identified in the analysis are equally used across newspapers.

## Supporting information

S1 Appendix Overview of studies on news media reporting about diabetes distinguishing period considered, region covered, analytical approach and focus (T1D, T2D or diabetes ‘in general’ – note that studies focusing on news media reporting of gestational diabetes exclusively are not included since it is not the focus of this paper). (DOCX)

S2 Appendix Collocates for ‘diabetes’ in the T1D corpus for grammatical patterns (frequency and collocation score in-between brackets). (DOCX)

S3 Appendix Collocates for ‘diabetes’ in the T2D corpus for grammatical patterns (frequency and collocation score in-between brackets). (DOCX)
