# Supplementary material for: Type 1 and Type 2 diabetes in the UK press: A diachronic corpus-based analysis
Source: PLoS One. 2026 Apr 30;21(4):e0348079. doi: 10.1371/journal.pone.0348079 (PMC13132212; doi:10.1371/journal.pone.0348079)
Supplement: S1 Appendix — (DOCX) [file pone.0348079.s001.docx]

**S1 Appendix.** Overview of studies on news media reporting about diabetes distinguishing period considered, region covered, analytical approach and focus (T1D, T2D or diabetes ‘in general’ – note that studies focusing on news media reporting of gestational diabetes exclusively are not included since it is not the focus of this paper).

| **Studies** | **Period** | **Context** | **Focus** | **Method** |
| --- | --- | --- | --- | --- |
| Bailey J, McCrossin T. Communicating diabetes in Australian print media: A change in language use be­tween 2010 and 2014? Australian and New Zealand Journal of Public Health. 2016;40(5):493–497. <https://doi.org/10.1111/1753-6405.12563> | 2010–2014 | Australia | Diabetes (in general) | Discourse analysis |
| Bednarek M. Invisible or high-risk: Computer-assisted discourse analysis of references to Aboriginal and Torres Strait Islander people(s) and issues in a newspaper corpus about diabetes. PLOS One. 2020;15(6):e0234486. | 2013–2017 | Australia | Diabetes (in general); Aboriginal and Torres Strait Islander people | Corpus-based discourse analysis |
| Bednarek M, Carr G. Diabetes coverage in Australian newspapers (2013‐2017): A computer‐based linguistic analysis. Health Promotion Journal of Australia. 2020;31(3): 497-503. | 2013–2017 | Australia | Diabetes (in general); Distinction of diabetes type 1 and type 2 | Corpus-based discourse analysis |
| Foley K, McNaughton D, Ward P. Monitoring the ‘diabetes epidemic’: A framing analysis of United Kingdom print news 1993-2013. PLOS One. 2020;15(1):e0225794 | 1993, 2001, 2013 | United Kingdom | Diabetes (in general) | Thematic analysis  Frame analysis |
| Gollust SE, Lantz PM. Communicating population health: print news media coverage of type 2 diabetes. Social Science & Medicine. 2009;69(7):1091-1098. | 2005–2006 | United States | Type 2 diabetes | Content analysis  Frame analysis |
| Gounder F, Ameer R. Defining diabetes and assigning responsibility: how print media frame diabetes in New Zealand. Journal of Applied Communication Research. 2018;46(1):93-112. | 2013–2014 | New Zealand | Type 1 diabetes;  Type 2 diabetes; Gestational diabetes | Content analysis  Frame analysis |
| Hellyer NE, Haddock-Fraser J. Reporting diet-related health issues through newspapers: portrayal of cardiovascular disease and Type 2 diabetes. *Health Education Research*. 2011;26(1):13-25. | 2008  (3 months) | United Kingdom | Cardiovascular disease;  Type 2 diabetes | Content analysis |
| Rock M. Diabetes portrayals in North American print media: a qualitative and quantitative analysis. American Journal of Public Health. 2005;95(10):1832-1838. | 1998–2000 (Canada news media)  1988–2001; 1991–2001 (US news media) | Canada  United States | Type 2 diabetes | Frame analysis |
| Santyarini R, Fajri MSA. The portrayal of diabetes in the Indonesian online press: a corpus-based discourse study. Cogent Arts & Humanities. 2024;11(1):2346374. | 2023 | Indonesia | Diabetes (in general) | Corpus-based discourse analysis |
| Stefanik-Sidener K. Nature, nurture, or that fast food hamburger: Media framing of diabetes in the New York Times from 2000 to 2010. Health Communication. 2013;28(4):351-358. | 2000–2010 | United States | Type 1 diabetes;  Type 2 diabetes;  Gestational diabetes | Content analysis  Frame analysis |
