# Supplementary material for: Type 1 and Type 2 diabetes in the UK press: A diachronic corpus-based analysis
Source: PLoS One. 2026 Apr 30;21(4):e0348079. doi: 10.1371/journal.pone.0348079 (PMC13132212; doi:10.1371/journal.pone.0348079)
Supplement: S2 Appendix — (DOCX) [file pone.0348079.s002.docx]

# **S2 Appendix**

Collocates for ‘diabetes’ in the T1D corpus for grammatical patterns (raw frequency and collocation score in-between brackets).

| **Grammatical pattern** | **T1D - year sub-corpora** | | | | |
| --- | --- | --- | --- | --- | --- |
|  | **2020** | **2021** | **2022** | **2023** | **2024** |
| *[i] Modifiers of 'diabetes'* | type 1 (20/12.5), Type 1 (5/10.9), undiagnosed (4/10.6), new-onset (4/10.6), type-2 (3/10.5), neonatal (3/10.4), careful (2/9.6), pregnancy (3/9.6), dementia (2/9.5), juvenile (2/9.4) | monogenic (22/13.1), maternal (6/11.4), gestational (6/11.3), type 1 (3/9.8), new-onset (2/9.7), juvenile (2/9.5), hospital (2/7.9), diabetes (2/5.8) | gestational (9/12.0), type 1 (6/10.9), Type 1 (4/10.8), uncontrolled (3/10.6), autoimmune (3/7.8) | type 1 (8/11.4), autoimmune (3/7.8) | Type 1 (7/12.0), type 1 (6/11.2) |
| *[ii] Verbs with 'diabetes' as object* | view (3/9.5), diagnose (5/8.8), tackle (2/8.4), present (2/8.3), cause (9/8.2), associate (2/8.2), develop (5/7.9), control (2/7.3), have (27/7.2), manage (2/7.2) | mistake (3/9.5), develop (12/9.1), trigger (4/8.9), pre-exist (2/8.8), diagnose (5/8.7), link (3/8.5), cause (8/8.0), have (36/7.6), include (7/7.6) | link (5/9.3), manage (7/9.0), delay (3/8.8), treat (4/8.5), diagnose (4/8.4), have (41/7.4), know (4/7.8), cause (4/7.0), get (5/6.6), be (6/4.2) | control (15/10.1), manage (15/10.0), associate (8/10.0), link (8/9.9), diagnose (4/8.4), have (36/7.6), develop (3/7.1), cause (4/7.0), be (4/3.7) | associate (21/11.2), manage (16/10.0), develop (11/8.9), control (6/8.6), link (3/8.3), monitor (3/8.0), cause (8/7.9), give (6/7.5), have (29/7.3) |
| *[iii] Verbs with 'diabetes' as subject* | control (4/9.8), mean (6/9.2), occur (3/9.0), demonstrate (2/8.8), tend (2/8.6), manage (2/8.4), affect (2/7.7), start (2/7.5), be (59/6.7), have (19/6.3) | destroy (4/9.5), mean (6/9.0), reverse (2/8.6), accelerate (2/8.6), start (5/8.6), grow (3/8.6), occur (2/8.1), affect (3/8.1), appear (2/7.7), lead (2/7.4), cause (3/7.4) | cause (13/9.4), emerge (4/9.1), change (4/8.7), affect (5/8.6), rely (3/8.6), occur (3/8.4), develop (4/8.3), need (4/7.8), lead (3/7.8), be (105/7.5) | manage (5/9.2), cause (10/9.0), tend (4/9.0), need (7/8.7), rise (3/8.3), mean (4/8.3), put (3/8.1), use (4/8.0), be (95/7.4), have (24/6.6) | occur (7/9.5), require (4/8.7), protect (3/8.6), tend (3/8.4), remain (4/8.4), appear (4/8.3), cause (6/8.2), be (128/7.8), need (4/7.8), begin (3/7.8) |
| *[iv] ‘Diabetes’ and/or…* | endocrinology (4/11.0), asthma (3/10.1), glaucoma (2/10.0), condition (4/9.2), arthritis (2/9.1), study (2/8.8), disease (5/8.6), level (2/8.4), cancer (2/8.3), type (2/8.3) | sclerosis (4/10.1), insufficiency (3/10.1), asthma (3/9.7), disease (11/9.7), change (3/9.5), epilepsy (2/9.3), pressure (3/9.3), obesity (3/9.2), lupus (2/9.1), type (4/9.1) | obesity (6/10.2), form (4/10.2), pancreas (3/9.5), condition (5/9.3), pressure (3/9.3), study (3/9.2), disease (7/9.0) | disease (21/10.5), condition (7/9.7), arthritis (4/9.7), depression (3/9.5), medicine (3/9.2), study (3/9.1) | disease (29/11.0), arthritis (5/9.8), screening (3/9.6), obesity (4/9.4), cancer (5/9.3), thyroid (3/9.2), type (4/8.9), scientist (3/8.9), condition (4/8.8), patient (3/8.4) |
| *[v] … with ‘diabetes’* | someone (2/10.4), adult (2/10.1), girl (1/9.6), people (12/9.5), population (1/9.5), everyone (1/9.5) diagnose (8/8.9), live (3/8.0), patient (1/7.9), child (1/7.4) | people (27/10.6), do (6/10.4), mother (3/10.1), patient (5/9.8), live (11/9.8), living (3/9.7), adult (2/9.4), diagnose (8/8.8), covid-19 (1/8.7), Briton (1/8.7) | people (40/11.1), live (17/10.3), struggle (6/10.2), human (3/9.9), adult (2/9.1), diagnose (9/9.0), living (2/8.9), patient (2/8.3), Americans (1/8.3), child (2/8.0) | person (4/10.9), struggle (7/10.8), live (13/10.1), people (13/9.6), living (2/9.5), good (1/9.2), pregnancy (1/9.1), someone (1/9.0), adult (1/8.7), child (2/8.6) | live (21/10.7), okay (4/10.6), people (20/10.2), adult (3/9.9), relative (2/9.6), everyone (2/9.5), mother (2/9.4), struggle (3/9.3), diagnose (10/9.1), patient (2/8.5) |
| *[vi] ‘diabetes’ is a…* | disease (6/12.1), condition (9/11.5) | challenge (5/12.0), disease (5/11.6), insulin (3/11.6), condition (8/11.2) | condition (20/12.4) | condition (10/11.6) | condition (14/12.0), disease (6/11.8) |
