# Supplementary material for: Type 1 and Type 2 diabetes in the UK press: A diachronic corpus-based analysis
Source: PLoS One. 2026 Apr 30;21(4):e0348079. doi: 10.1371/journal.pone.0348079 (PMC13132212; doi:10.1371/journal.pone.0348079)
Supplement: S3 Appendix — (DOCX) [file pone.0348079.s003.docx]

# **S3 Appendix**

Collocates for ‘diabetes’ in the T2D corpus for grammatical patterns (raw frequency and collocation score in-between brackets).

| **Grammatical pattern** | **T2D - year sub-corpora** | | | | |
| --- | --- | --- | --- | --- | --- |
|  | **2020** | **2021** | **2022** | **2023** | **2024** |
| *[i] Modifiers of 'diabetes'* | type-2 (70/12.4), disease (45/10.9), gestational (16/10.5), Type-2 (14/10.3), undiagnosed (12/10.0), type-1 (10/9.9), adult (11/9.9), full-blown (9/9.7), uncontrolled (8/9.5), obesity (17/9.5) | gestational (89/12.8), type-2 (48/12.1), monogenic (18/10.8), Type-2 (10/10.0) Lancet (9/9.7), full-blown (7/9.4), obesity (12/9.2), cancer (7/8.6), pressure (6/8.6), maternal (4/8.6) | gestational (25/11.7), type-2 (22/11.5), disease (23/10.3), uncontrolled (9/10.3), obesity (23/10.0), undiagnosed (7/9.8), Type-2 (4/9.2), pressure (5/8.7), cancer (4/8.0), diabetes (6/7.3) | gestational (49/12.2), type-2 (36/11.8), Lancet (8/9.6), disease (17/9.5), pressure (11/9.3), Type-2 (6/9.3), cancer (13/9.2), stroke (7/9.1), uncontrolled (5/9.0), obesity (13/8.7) | type-2 (98/12.2), gestational (47/11.3), Lancet (43/11.0), disease (62/10.4), Type-2 (20/10.2), cancer (29/9.3), uncontrolled (10/9.2), pressure (14/8.9), obesity (20/8.7), undiagnosed (7/8.6) |
| *[ii] Verbs with 'diabetes' as object* | develop (86/10.6), reverse (40/10.2), have (212/9.9), diagnose (20/9.2), put (25/9.1), compare (16/8.8), get (29/8.7), stop (14/8.6), keep (16/8.4), link (12/8.4) | reverse (41/10.7), develop (46/10.0), have (136/9.5), diagnose (17/9.5), manage (17/9.5), treat (18/9.3), associate (13/8.9), cause (20/8.8), miss (7/8.4), control (8/8.3), trigger (7/8.2) | reverse (35/10.6), develop (55/10.4), have (55/10.4), treat (18/9.4), manage (14/9.2), think (12/8.9), diagnose (10/8.8), link (12/8.8), prevent (11/8.6), include (13/7.9) | treat (49/10.3), develop (69/10.3), reverse (23/9.9), manage (29/9.9), have (130/9.2), control (17/9.2), link (17/8.9), put (16/8.8), compare (11/8.5), cause (20/8.4) | treat (95/10.6), reverse (54/10.4), develop (109/10.3), manage (43/9.8), control (19/8.8), have (158/8.7), compare (20/8.6), diagnose (15/8.5), put (17/8.2), link (16/8.0) |
| *[iii] Verbs with 'diabetes' as subject* | be (466/9.5), go (30/9.4), occur (18/9.1), have (100/8.6), develop (13/8.6), face (12/8.5), double (11/8.5), cause (14/8.4), increase (13/8.4), put (11/8.3) | increase (16/9.3), occur (13/9.3), double (12/9.1), be (303/9.0), go (15/8.8), affect (10/8.6), develop (9/8.5), have (80/8.3), cause (10/8.3), rise (7/8.2) | be (354/9.5), cause (21/9.3), have (99/9.1), occur (10/8.8), develop (10/8.6), affect (10/8.6), include (13/8.4), rise (6/7.9), lose (6/7.7), increase (5/7.5) | cause (25/9.5), occur (13/9.1), be (324/8.9), affect (12/8.8), remain (10/8.7), use (12/8.3), cost (6/8.0), have (64/8.0), increase (7/7.9), need (7/7.8) | occur (20/9.2), affect (18/8.8), be (469/8.6), take (20/8.2), rise (11/8.1), cause (14/8.0), have (115/8.0), double (8/8.0), remain (9/7.8), go (13/7.7) |
| *[iv] ‘Diabetes’ and/or…* | disease (266/11.9), pressure (117/11.2), obesity (96/10.9), cancer (84/10.7), stroke (38/9.6), hypertension (27/9.5), problem (29/9.5), study (23/9.0), dementia (20/8.9), condition (16/8.6) | disease (162/11.5), obesity (87/11.2), cancer (87/11.1), pressure (74/10.8), condition (21/9.3), hypertension (15/9.0), dementia (16/9.0), cholesterol (15/8.9), stroke (17/8.7), attack (14/8.6) | disease (246/11.9), pressure (85/10.9), obesity (74/10.8), cancer (73/10.7), condition (26/9.6), hypertension (21/9.4), study (24/9.3), research (18/9.1), dementia (17/9.0), prediabetes (14/8.8) | disease (301/11.8), cancer (130/11.1), obesity (95/10.7), pressure (82/10.5), condition (26/9.2), stroke (34/9.0), depression (23/8.9), study (24/8.9), research (19/8.7), problem (19/8.6) | cancer (256/11.1), pressure (117/10.1), dementia (50/9.2), hypertension (45/9.2), condition (46/9.1), stroke (57/9.0), study (42/8.8), Wegovy (37/8.8), cholesterol (36/8.7), attack (36/8.6) |
| *[v] … with ‘diabetes’* | people (126/12.1), live (26/10.8), diagnose (29/10.7), someone (9/10.0), patient (13/9.6), individual (7/9.6), man (6/9.2), UK (5/9.2), adult (5/9.1) | diagnose (64/11.9), people (78/11.9), live (36/11.6), pregnant (9/10.1), patient (7/9.2), mother (4/9.0), adult (4/9.0), woman (4/8.8), child (3/8.4), pet (2/8.1) | people (83/11.9), diagnose (33/11.3), live (26/11.1), adult (7/9.8), patient (7/9.5), struggle (5/9.3), human (4/9.3), woman (5/9.3), living (3/8.7), depression (2/8.3) | live (59/11.8), people (89/11.5), patient (28/10.8), diagnose (20/10.2), woman (9/9.8), adult (8/9.6), struggle (7/9.3), living (4/8.8), anyone (4/8.8), link (3/8.5) | people (149/11.8), live (84/11.6), diagnose (39/10.4), adult (18/10.2), patient (24/10.1), relative (10/9.6), individual (7/9.0), living (5/8.6), battle (5/8.5), person (4/8.3) |
| *[vi] ‘diabetes’ is a…* | condition (24/12.3), disease (13/11.5), form (10/11.3), crisis (7/10.9), time (13/10.9), factor (9/10.2) | condition (22/12.9), disease (14/12.3) | condition (48/13.2), time (10/11.1), disease (6/10.7), form (4/10.2) | condition (22/12.6), form (5/11.0), disease (5/10.8) | condition (42/12.9), disease (13/11.4), problem (5/10.1), factor (5/9.5) |
